# *Stenotrophomonas maltophilia* virulence: a current view

**DOI:** 10.3389/fmicb.2024.1385631

**Published:** 2024-04-29

**Authors:** Vladimir Mikhailovich, Rustam Heydarov, Danila Zimenkov, Igor Chebotar

**Affiliations:** ^1^Engelhardt Institute of Molecular Biology, Russian Academy of Sciences, Moscow, Russia; ^2^Pirogov Russian National Research Medical University, Moscow, Russia

**Keywords:** *Stenotrophomonas maltophilia*, virulence factors, biofilms, quorum sensing, iron uptake systems

## Abstract

*Stenotrophomonas maltophilia* is an opportunistic pathogen intrinsically resistant to multiple and broad-spectrum antibiotics. Although the bacterium is considered a low-virulence pathogen, it can cause various severe diseases and contributes significantly to the pathogenesis of multibacterial infections. During the COVID-19 pandemic, *S. maltophilia* has been recognized as one of the most common causative agents of respiratory co-infections and bacteremia in critically ill COVID-19 patients. The high ability to adapt to unfavorable environments and new habitat niches, as well as the sophisticated switching of metabolic pathways, are unique mechanisms that attract the attention of clinical researchers and experts studying the fundamental basis of virulence. In this review, we have summarized the current knowledge on the molecular aspects of *S. maltophilia* virulence and putative virulence factors, partially touched on interspecific bacterial interactions and iron uptake systems in the context of virulence, and have not addressed antibiotic resistance.

## Introduction

The global emergence of multidrug-resistant Gram-negative bacteria is the most challenging clinical and public health problem (Hernando-Amado et al., 2019; Antimicrobial Resistance Collaborators, 2022). Despite significant progress in biomedical research, many untreatable infectious diseases are considered the leading causes of human death worldwide. Nosocomial and community-acquired infections caused by opportunistic Gram-negative pathogens are becoming increasingly difficult to treat as both intrinsic and acquired antibiotic resistance has increased significantly in recent years (Theuretzbacher et al., 2020).

Among Gram-negative opportunistic pathogens, *Stenotrophomonas maltophilia* has been the subject of an increased interest and extensive research over the last two decades. The number of reported *S. maltophilia* infections has considerably risen and the bacterium has been classified as the most common Gram-negative carbapenem-resistant pathogen in patients with bacteremia in some US hospitals (Cai et al., 2020).

*Stenotrophomonas maltophilia* is a globally dispersed, non-fermenting Gram-negative bacillus frequently isolated in the environment, particularly from water sources, soil, sediment, plants, and animal specimens (Aznar et al., 1992; Nakatsu et al., 1995; Jakobi et al., 1996; Berg et al., 1999; Johnson et al., 2003; Ivanov et al., 2005; Romanenko et al., 2008; Berg, 2009). According to the List of Prokaryotic Names with Standing in Nomenclature,^1^ the genus *Stenotrophomonas* comprises at least 25 validated species exhibiting great genetic diversity and metabolic heterogeneity both within the *Stenotrophomonas* genus and within a single species (Ryan et al., 2009; Turrientes et al., 2010; Pompilio et al., 2016). Comprehensive taxonomic and phylogenomic studies have shown that *S. maltophilia* includes multiple cryptic species, forming the *Stenotrophomonas maltophilia* complex (Smc) and a distinction that defies conventional classification approaches (Ochoa-Sánchez and Vinuesa, 2017; Kumar et al., 2020; Singh et al., 2023). Identification of phylogenetic relationships among *Stenotrophomonas* spp. based on analysis of core protein sequences revealed 24 species-level clades of Smc (Gröschel et al., 2020; Li et al., 2024).

The bacterium demonstrates high adaptability to various environments, including nutrient-limited and hostile conditions. *S. maltophilia* is capable of utilizing a wide range of carbon sources such as trichloroethylene, toluene, chloroform, glucose, and benzene (Lee et al., 2002; Pompilio et al., 2011; Mukherjee and Roy, 2013).

*Stenotrophomonas maltophilia* is an opportunistic pathogen intrinsically resistant to multiple and broad-spectrum antibiotics. The bacterium is associated with a number of serious diseases and contributes significantly to the pathogenesis of multibacterial infections. *S. maltophilia* causes infections in various human organs, including the respiratory, gastrointestinal, and urinary tracts. It can cause severe pneumonia, catheter-associated bacteremia/septicemia, osteochondritis, mastoiditis, meningitis, and endocarditis (Denton and Kerr, 1998; Brooke, 2012; Chang et al., 2015). The bacterium is frequently recovered in the lungs of cystic fibrosis (CF) patients; according to various studies, the frequency ranges from 10% to 30% (Waters et al., 2011; Cuthbertson et al., 2020). During the global COVID-19 pandemic, *S. maltophilia* has been recognized as one of the most common causative agents of respiratory co-infections and bacteremia in critically ill COVID-19 patients. Furthermore, *S. maltophilia* isolates detected in sputum samples obtained from these patients had the highest rates of multidrug resistance among other bacteria infecting COVID-19 patients (Yang S. et al., 2021; Ishikawa et al., 2022; Langford et al., 2023).

*Stenotrophomonas maltophilia* is also of interest as an active member of polymicrobial bacterial communities: it can influence the metabolism of neighboring microorganisms, either through antagonistic suppression of other species or by symbiotic coexistence. A vivid example of such inter-species communication can be observed in CF patients, where *S. maltophilia* colonizes the host along with other major pathogens, such as *Pseudomonas aeruginosa*, *Staphylococcus aureus*, nontuberculous mycobacteria, or *Burkholderia cenocepacia* (Goss et al., 2004; Coutinho et al., 2008).

The pathogenesis of infections caused by *S. maltophilia* is determined by numerous virulence factors (VFs), molecules that facilitate bacterial colonization of the host at the cellular level, thereby initiating the infectious process. *S. maltophilia* possesses a considerable spectrum of VFs or putative factors associated with virulence. These factors include surface cell-associated structures (lipopolysaccharides, type IV pili, flagella, fimbriae, and nonpilus adhesins); the production of a wide spectrum of extracellular enzymes (e.g., proteases, esterases, lipases), hemolysin, siderophores, and cytotoxins; the ability to form biofilms on abiotic surfaces and host tissues; SmeYZ, SmeDEF, SbiAB, and MacABCsm efflux pumps; and the secretion of small molecules in the environment via Quorum Sensing (QS) intercellular communication system [the diffusible signal factor (DSF) and outer membrane vesicles (OMV); Looney, 2005; Brooke, 2012; Ferrer-Navarro et al., 2013; Abbott and Peleg, 2015; Trifonova and Strateva, 2019; Wu C.-J. et al., 2022].

In this review, we briefly summarize the current knowledge on *S. maltophilia* virulence and provide an overview of the literature introducing the virulence determinants and their regulation in *S. maltophilia*. We have limited the scope of the review to virulence, partially touching upon inter-species bacterial interactions and iron uptake systems in the context of virulence, and have not referred to antibiotic resistance.

## Adhesins as virulence factors

Adherence of the bacterium to host tissues is a crucial step in the host-pathogen interaction. At this stage, the pathogen attached to the host cell initiates its own biochemical processes aimed at its proliferation, invasion of host cells, secretion of toxic molecules, and activation of host cell signaling cascades.

**Figure 1:**
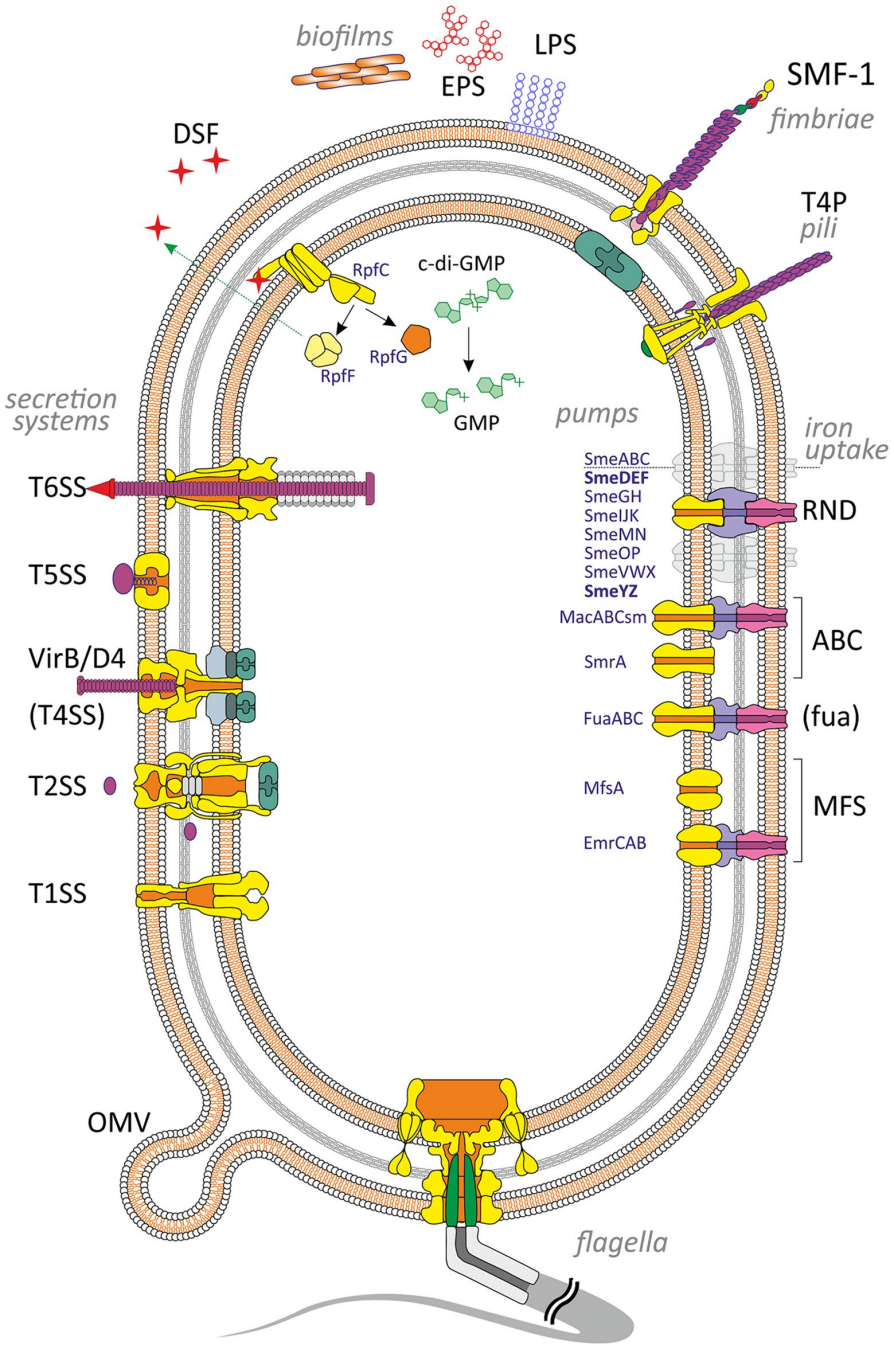
Virulence and putative virulence factors in *Stenotrophomonas maltophilia*. Surface cell-associated structures include lipopolysaccharides (LPS), type IV pili (T4P), flagella, fimbriae (SMF-1), and non-pilus adhesins (not shown). Extracellular enzymes are secreted through type I, II, IV, V, and VI secretion systems. Small molecules efflux to the environment via the diffusible signal factor (DSF) and outer membrane vesicles (OMV). *S. maltophilia* produces extracellular polymeric substances and forms a self-secreted polymeric matrix, biofilms, consisting of exopolysaccharides (EPS), DNA, and proteins. The intracellular c-di-GMP level contributes to numerous virulence factors (see the text for details). Different types of efflux pumps revealed in *S. maltophilia* are shown on the right. The pumps involved in virulence are marked in bold.

Bacterial adherence factors, also known as adhesins, are polypeptides or polysaccharides. Protein adhesins are cell-surface components or appendages that can be divided into two groups: fimbrial and afimbrial. Polysaccharide adhesins are generally associated with the bacterial cell wall, outer membrane, or capsule. It should be noted that adhesion functions in pathogenesis are not limited to the initial host-pathogen interaction; adhesins also play a significant role in subsequent stages of infection (see below). To provide a holistic view of the role of various VFs in the development of *S. maltophilia* infection, cell-associated and extracellular VFs are summarized and illustrated in Figure 1.

## Cell-surface polysaccharides

*Stenotrophomonas maltophilia* has lipopolysaccharides (LPS) comprising lipid A, core oligosaccharide, and O-antigen (Neal and Wilkinson, 1982; Jucker et al., 1996; Zhang and Kong, 2002; McKay et al., 2003). LPS is a robust inducer of TNF-α production by macrophages due to its lipid A moiety, that has been elegantly demonstrated by Waters et al. (2007) in a mouse model. Despite the relatively weak invasiveness of *S. maltophilia*, the level of TNF-α production after stimulating cells of macrophage cell line RAW for 4 h with purified lipid A isolated from *S. maltophilia* was significantly higher than the corresponding level obtained after stimulation with lipid A from the laboratory *P. aeruginosa* PAO1 control strain (Waters et al., 2007).

Core oligosaccharides play an essential role in LPS formation and therefore in virulence. Defects in core oligosaccharides of certain bacteria (e.g., *P. aeruginosa*, *Bordetella bronchiseptica*) are associated with decreased virulence or the emergence of avirulent strains (Goldberg et al., 1995; West et al., 2000).

The contribution of O-antigens to virulence has also been reported to be significant for many bacteria. Bacteria lacking O-antigens or possessing defective antigen molecules due to disruptions in biosynthesis may exhibit decreased virulence properties. This phenomenon has been demonstrated, in particular, for the species *Burkholderia pseudomallei, P. aeruginosa,* and *B. abortus* (Goldberg et al., 1995; DeShazer et al., 1998; Ugalde et al., 2000). In *S. maltophilia*, defective LPS lacking the O-antigen can affect biofilm production, twitching motility (the ability to move on surfaces using type IV pili), and swimming motility (Huang et al., 2006; Brooke et al., 2008). *Stenotrophomonas maltophilia* lipopolysaccharides show structural diversity between strains within the genus, with at least 31 different O-antigens (Winn and Wilkinson, 2001; Waters et al., 2007).

The metabolic process of carbohydrates and their incorporation into LPS in *S. maltophilia* is regulated by several genes. The *spgM* gene involved in this process encodes a bifunctional enzyme with both phosphoglucomutase and phosphomannomutase activity. This gene is homologous to *algC* which controls alginate and LPS biosynthesis in *P. aeruginosa* (McKay et al., 2003; Flores-Treviño et al., 2019). McKay et al. (2003) have shown that *spgM* mutants with shorter O-polysaccharide chains lost virulence in the rat lung model and exhibited increased susceptibility to complement-mediated killing.

Two operons, *rmlBACD* and *xanAB*, are involved in the synthesis of polysaccharides in *S. maltophilia*. Huang et al. (2006) have conducted an SDS-PAGE analysis of purified LPS from *S. maltophilia rmlA*, *rmlC*, and *xanB* mutants and found these genes necessary for LPS O-antigen biosynthesis. In addition, *xanB* is required for the production of the LPS core region. The authors also suggested that *rml* and *xanB* are involved in the biosynthesis of exopolysaccharides (EPS): the *rmlA* and *rmlC* mutants exhibited decreased biofilm production on polystyrene and increased biofilm density on glass. Meanwhile, the *xanB* mutant displayed lower biofilm production only on polystyrene. Besides contributing to biofilm formation, alterations in LPS caused by the *rmlAC* and *xanB* mutations, may lead to changes in outer membrane appendages, such as flagella and type IV pili, thereby interfering with motility and attachment (Huang et al., 2006).

## Flagella

*Stenotrophomonas maltophilia* possesses one or multiple polar flagella that confer swimming motility, swarming and chemotaxis. Flagella facilitate primary adherence to biotic and abiotic surfaces, contribute to colonization and invasion in the early stages of infection, and trigger specific immune responses by host cells (de Oliveira-Garcia et al., 2002; Huang et al., 2006; Pompilio et al., 2010, 2018; Zgair and Chhibber, 2011).

Wu et al. have identified three flagellar genes, *fliC1*, *fliC2*, and *fliC3* which form an operon in the sequenced *S. maltophilia* genome. The authors generated single, double, and triple mutants corresponding to these genes and revealed that each gene contributed to swimming, adhesion, and biofilm formation. The ability to attach, swim, and form biofilms decreased proportionally to the number of deactivated genes, with the triple mutant losing its swimming ability and significantly compromising adhesion and biofilm formation. Thus, flagella in motile pathogens can be considered important VFs (Moens and Vanderleyden, 1996; Duan et al., 2013; Wu C.-J. et al., 2022).

Pompilio et al. (2010) have constructed two *S. maltophilia* mutants in which the *fliI* gene was inactivated. The highly conserved *fliI* gene encodes a substrate-specific ATPase (FliI) that provides energy for the active translocation of flagellar structural components in a wide range of bacterial species (Yonekura et al., 2002; Di Bonaventura et al., 2007). These two flagellum-deficient *S. maltophilia fliI* mutants exhibited decreased adherence to CF-derived bronchial epithelial IB3-1 cells and compromised swimming motility (Pompilio et al., 2010).

Inbred BALB/c mice (commonly used as animal models for drug and vaccine testing) pretreated intranasally with purified *S. maltophilia* flagellin and instilled with *S. maltophilia* 4 h later exhibited significantly increased levels of pro-inflammatory cytokines IL-1β and TNF-α, myeloperoxidase activity, caspase-1 activity, and nitric oxide compared to control groups (Zgair and Chhibber, 2012). The pretreated mice also demonstrated elevated levels of neutrophils, lymphocytes, and monocytes in their bronchoalveolar lavage fluid providing nonspecific protection for the animals against *S. maltophilia* as well as *S. aureus* infections (Zgair and Chhibber, 2012). In another study, Pompilio et al. (2018) compared the severity of disease caused by aerosol challenge of mice of the DBA/2 N strain with the clinical isolate *S. maltophilia* SM111 and its aflagellate isogenic mutant (Δ*fliI*). Pompilio et al. (2018) did not observe a significant trend in body weight changes, pulmonary persistence, lung damage, or mortality in mice infected with wild-type and *fliI^−^* strains. The authors suggested that flagella and motility might not represent *S. maltophilia* virulence traits involved in the pathogenesis of lung infection. Meanwhile, the expression of TNF-α in murine lungs infected with the aflagellate mutant was significantly reduced (Pompilio et al., 2018).

Hypothetically, in the context of a chronic infection, it is admissible that a bacterium lacking flagellin as a significant immunogenic factor may gain a survival advantage diminishing the host’s immune response. This hypothesis could be corroborated by observations in patients with CF: a substantial portion of *P. aeruginosa* isolates (39%) from chronically colonized patients were nonmotile and resistant to phagocytosis by macrophages (Mahenthiralingam et al., 1994). Nevertheless, the majority of studies have reported a positive correlation between motility and primary adhesion (e.g., de Oliveira-Garcia et al., 2002; Waters et al., 2007; Pompilio et al., 2010; Zgair and Chhibber, 2011; Madi et al., 2016). Perhaps, the aforementioned speculation that flagella do not contribute to *S. maltophilia* virulence may be relevant only to the later (chronic) stage of the disease when the initial adhesion step preceding the infection has already occurred.

The motility of *S. maltophilia* and the level of flagellin gene expression depend on environmental conditions and are tightly controlled by comprehensive genetic systems that have not been fully investigated. In particular, cyclic diguanosine monophosphate (c-di-GMP), the ubiquitous second messenger has been recognized as an important intracellular signaling molecule involved in the regulation of flagellin gene expression in many bacteria. c-di-GMP is also implicated in controlling various bacterial physiological processes, including the cell cycle, adherence, motility, the production of VFs, and biofilm formation (Ross et al., 1987; Hengge, 2009).

The intracellular concentration of c-di-GMP is modulated by two groups of enzymes with opposing activities, diguanylate cyclases (DGCs) and phosphodiesterases (PDEs). DGCs contain a conserved GGDEF domain and synthesize c-di-GMP by condensation of two GTP molecules. PDEs, with either EAL or HD-GYP domains, degrade c-di-GMP into linear guanosine dinucleotide (pGpG) or guanosine monophosphate (GMP) (Chan et al., 2004; Christen et al., 2005, 2006; Caly et al., 2014). An increased level of c-di-GMP, resulting from the action of DGCs, is associated with a sessile lifestyle and biofilm formation which is relevant to chronic bacterial infections. Conversely, lower concentrations of c-di-GMP due to phosphodiesterase activity, are found in motile bacteria during acute infection processes (Hengge, 2009; Moscoso et al., 2011; Cheng et al., 2019). In *S. maltophilia*, the mechanisms by which c-di-GMP controls flagellar synthesis and flagella numbers are still poorly understood, and there is a paucity of studies focused on the molecular basis of its functioning.

In some bacteria, the expression of flagellar genes is activated and controlled by specific genetic determinants known as master regulators and their homologs, e.g., FlrA (FleQ) in *P. aeruginosa* and *Vibrio cholerae*, flaK and flaM in *Vibrio parahaemolyticus* (Ritchings et al., 1995; Stewart and McCarter, 1996; Arora et al., 1997; Klose and Mekalanos, 1998; Hickman and Harwood, 2008).

Yang et al. (2014) reported that flagellar gene expression in *S. maltophilia* is controlled by FleQ (Smlt2295). This transcriptional regulator, an enhancer-binding protein, is homologous to the similar master regulator in *P. aeruginosa* and it is the major target for the c-di-GMP produced by the wrinkly spreader phenotype (Wsp) chemosensory system pathway (Hickman et al., 2005; Yang et al., 2014). FleQ works together with a putative ATPase, FleN, and is inhibited by binding c-di-GMP. Inhibition of this complex and, therefore, Wsp pathway activation results in elevated expression of biofilm-associated *pel*, *psl,* and *cdr* operons and a reduction of flagellar gene expression (Hickman et al., 2005; Hickman and Harwood, 2008; Yang et al., 2014). The situation is reversed if the concentration of c-di-GMP is low: FleQ remains unbounded, and it leads to increased expression of flagellar genes, and, therefore, the ability of bacteria to be sessile in biofilms decreases.

Liu et al. (2017) have reported a correlation between increased expression of *bsmR* which encodes an eponymous regulatory protein, an EAL domain-containing PDE, and increased bacterial swimming and decreased cell aggregation in *S. maltophilia* CGMCC 1.1788. Therefore, *bsmR* is suggested to be a negative regulator of biofilm formation and a positive regulator of swimming motility. The *bsmR* operon controls the expression of at least 349 genes, of which 34 are involved in flagellar synthesis and are under positive regulation of the FsnR transcription factor (Liu et al., 2017). BsmR degrades c-di-GMP to activate the expression of FsnR. This flagellar-assembly-related transcription factor binds directly to the promoter regions of two operons, *Smlt2303* and *Smlt2318*, initiating the transcription of flagella-associated genes (Kang et al., 2015; Zheng et al., 2016).

Zhang et al. (2022) analyzed genes potentially affecting the c-di-GMP level in *S. maltophilia*, specifically those encoding proteins containing GGDEF, EAL, and HD-GYP domains. The authors identified 33 putative c-di-GMP turnover enzymes in the genome of *S. maltophilia* using the Simple Modular Architecture Research Tool (SMART) (Letunic and Bork, 2018) and constructed mutants of all 33 genes via insertional inactivation. Among the mutants analyzed, 12 bacterial strains exhibited a deficiency in swimming motility while one showed promotion, that suggests the 13 corresponding genes may contribute to the regulation of bacterial swimming motility. The authors also made an important observation that the mutation-induced degeneration or inactivation of DGCs or PDEs do not necessarily alter the cellular c-di-GMP level and bacterial swimming motility. Therefore, further investigations are needed to assess the contribution of each gene to swimming motility.

Among all the enzymes analyzed, the authors also identified and characterized a novel Fe^2+^-dependent phosphodiesterase named SisP (*S. maltophilia* iron-sensing PDE). SisP increased its activity and facilitated bacterial swimming upon stimulation with ferrous iron in a dose-dependent manner, and the degradation of c-di-GMP led to FsnR-dependent transcription of flagellar genes (Zhang et al., 2022).

## Fimbriae and pili

Type 1 fimbriae (SMF-1) are an important VF that confers to *S. maltophilia* the ability to adhere to various specific host epithelia (de Oliveira-Garcia et al., 2002; Zgair and Chhibber, 2011; Giltner et al., 2012). In particular, it has been reported that adherence to biotic (epithelial cells) and abiotic surfaces (such as medical implants and catheters) was inhibited by anti-SMF-1 antibodies (de Oliveira-Garcia et al., 2003). Fimbriae are also involved in early stages of biofilm formation (de Oliveira-Garcia et al., 2003) and can agglutinate red blood cells (Crossman et al., 2008). Antibodies against SMF-1 fimbriae, but not preimmune serum, inhibited hemagglutination in a dose-dependent manner (de Oliveira-Garcia et al., 2003).

It is worth noting that SMF-1 fimbriae were revealed in all clinical isolates (*n* = 46) obtained from patients (de Oliveira-Garcia et al., 2003), whereas *S. maltophilia* strains isolated from environmental sources did not possess them (Nicoletti et al., 2011). Therefore, fimbriae are thought to be the significant structures involved in the adhesion and colonization of the lung epithelium.

Fimbrial protein production is controlled by the *Smf-1* fimbrial operon, which includes Smlt0706-Smlt0709 (Crossman et al., 2008). This type of fimbriae is assembled by the bacterial chaperone-usher pathway (Thanassi et al., 1998). Despite *S. maltophilia* fimbrin is closely related to fimbrial adhesins of most members of the *Enterobacteriaceae* and the CupA fimbriae of *P. aeruginosa*, the N-terminal amino acid sequences of *S. maltophilia* Smf-1 significantly differ from those belonging to other families of fimbriae (ranging from 50% to 61%). This suggests that this family of fimbriae may extend to other genetically distant non-enteric bacterial genera (Vallet et al., 2001; de Oliveira-Garcia et al., 2003).

Type IV pili (T4P) are considered a significant VF associated with twitching motility, adhesion to biotic and abiotic surfaces, colonization, and biofilm formation in various bacterial pathogens (Doig et al., 1988; Saiman et al., 1990; Giltner et al., 2012). T4P have also been reported to mediate *P. aeruginosa* virulence through interdependent action with the type III secretion system (T3SS), thereby promoting its effector injection into the host cell (Heiniger et al., 2010; Shikata et al., 2016).

To date, the role of type IV pili in *S. maltophilia* virulence has not been sufficiently studied. Kalidasan and Neela (2020) have proposed a theoretical model for *S. maltophilia* type IV pilus based on Rapid Annotations using Subsystem Technology (RAST) analysis that provided high quality genome annotations for bacterial genomes across the whole phylogenetic tree, and previous reports on *P. aeruginosa*.

Extensive sequence variation in the type IV pilin adhesion precursor gene has been revealed by Fluit et al. (2022). Meanwhile, no significant correlations have been reported between virulence and the presence of the *pil* gene family which is involved in pilus formation. An analysis of clinical and environmental *S. maltophilia* strains performed by Cruz-Córdova et al. (2020) showed that the *pilU* gene frequencies were high enough but comparable in both groups analyzed, regardless of origin. An increase in biofilm biomass formed by CF isolates with elevated swimming and twitching motility has been reported by Pompilio et al. (2011) and, notably, the phenomenon was observed only in CF isolates. Taken together, there appears to be no direct evidence for T4P as a significant VF in *S. maltophilia*, and further studies are needed to clarify their contribution to its virulence.

## Secretion systems and extracellular enzymes

Clinical *S. maltophilia* strains produce a variety of VFs, including proteases (StmPr1, StmPr2, StmPr3, StmPr4), lipases (lipase and phospholipase C and D), nucleases, gelatinases, elastase, esterases, hyaluronidases, fibrinolysin/streptokinase, heparinases, hemolysins, siderophores, and cytotoxins (Windhorst et al., 2002; Travassos et al., 2004; Trifonova and Strateva, 2019). These VFs contribute to bacterial colonization/persistence, induce cytotoxic and morphological effects on host cells, and play roles in various stages of the infection process (Karaba et al., 2013; DuMont et al., 2015).

Of the 11 known bacterial secretion systems (including outer membrane vesicles, OMVs), *S. maltophilia* possesses type I, II, IV, V, and VI secretion systems that have been identified through genome sequencing (Crossman et al., 2008; Rocco et al., 2009; Zhu et al., 2012; Adamek et al., 2014; Alavi et al., 2014). Albeit the role of these systems in virulence formation is well understood in many bacteria, only three types of *S. maltophilia* secretion systems (II, IV, and VI) have been described in detail.

The genome of *S. maltophilia* clinical strain K279a has two unlinked loci that are predicted to encode the double membrane-spanning type II secretion system, T2SS (GSP and XPS). Each locus contains 11 T2SS genes, corresponding to the core T2SS components (Karaba et al., 2013). The *S. maltophilia* type II secretion system mediates the secretion of at least seven protein effectors and three proteolytic activities. Proteolytic enzymes, particularly the serine proteases StmPr1, StmPr2, and StmPr3, are secreted in an XPS-dependent manner and induce structural and viability changes in lung epithelial cells, promoting the degradation of collagen, fibrinogen, fibronectin, and interleukin 8 (IL-8) (Karaba et al., 2013; DuMont et al., 2015; DuMont and Cianciotto, 2017). Another serine protease, StmPR4, has also been reported in the *S. maltophilia* genome (Windhorst et al., 2002; Ribitsch et al., 2012). It is thought that StmPR3, together with StmPR1 and StmPR2, contributes to the protease-mediated dysfunction of the innate immune system in cystic fibrosis (Molloy et al., 2019).

Lee et al. (2022) purified and identified a serine colistin-degrading protease (Cdp) in *S. maltophilia* strain Col1. Isolated from the soil, this strain exhibited high-level resistance against colistin (MIC value of 32 mg/L). Coculture and coinfection assays revealed that *S. maltophilia* strain Col1, bearing the *cdp* gene, could inactivate colistin, thereby protecting susceptible *P. aeruginosa*. Using colistin against *P. aeruginosa* infection in *Drosophila melanogaster* increased fly survival by 41%. In contrast, coinfection of flies with *S. maltophilia* strains carrying the *cdp* gene, did not increase the survival rate after colistin treatment. The authors noted that *S. maltophilia* genomes contain genes orthologous to *cdp*, located in a region immediately adjacent to the T2SS gene cluster (Lee et al., 2022). Thus, the colistin-degrading protease may play an important role in collective resistance to colistin in polymicrobial infections such as CF (Lee et al., 2022).

A type IV secretion system (T4SS) has been identified in the genome of both clinical and environmental *S. maltophilia* isolates (Nas et al., 2019). In *S. maltophilia*, the T4SS called the VirB/D4 system, is highly conserved within the genus and it is most similar to the T4SS of the *Xanthomonas* genus (Nas et al., 2019). T4SS typically comprises 12 proteins (VirB1-VirB11, and VirD4; Christie et al., 2014; Ghosal et al., 2017; Grohmann et al., 2018) and facilitates the delivery of DNA and/or protein effectors into bacterial or eukaryotic targets in a contact-dependent manner (Gonzalez-Rivera et al., 2016; Grohmann et al., 2018). The VirB10 protein, as part of the periplasm-outer membrane-spanning subcomplex, and the ATPase coupling protein VirD4 are essential for the antibacterial activity of the T4SS in *S. maltophilia* K279a (Bayer-Santos et al., 2019; Nas et al., 2019, 2021). The contribution of the VirB/D4 system to interspecific antagonism was elegantly demonstrated by Nas et al. (2019). They reported that *S. maltophilia* was capable of killing *P. aeruginosa* environmental strain 7700 and clinical isolates PAO1 and PAK when cocultured. Interestingly, *S. maltophilia* exhibited selectivity when acting on different species of heterologous bacteria, e.g., it killed *Pseudomonas mendocina* but not *P. fluorescens*, *P. putida*, or *P. stutzeri* (Nas et al., 2019). Based on studies that highlight the contribution of the type VI secretion systems in various bacteria to interbacterial killing (Basler et al., 2012; Ma et al., 2014; Ahmad et al., 2019), the authors suggested that the *S. maltophilia* VirB/D4 T4SS effectors are akin to those secreted by other type VI secretion systems, e.g., lipases, peptidases, nucleases, and muramidases (Ho et al., 2014).

Nas et al. (2021) identified 13 putative cognate immunity proteins in *S. maltophilia* that typically provide self-protection to the organism encoding the T4SS, and studied the effect of their expression in heterologous bacteria. Using these proteins, the authors revealed two potential antibacterial effectors, RS14245 and RS14255, that were required for the ability of *S. maltophilia* to kill heterologous bacteria, especially laboratory *E. coli* and clinical strains of *P. aeruginosa* isolated from the lungs of CF patients. The putative lipases, RS14245 and RS14255, when bound by cognate immunity proteins, did not exhibit antibacterial activity; and *S. maltophilia* complemented mutants lacking RS14245 and RS14255 significantly reduced their antibacterial properties (Nas et al., 2021).

The authors’ findings are intriguing from various perspectives. On the one hand, the secretion of effectors that suppress other bacterial species can be regarded as a significant VF of *S. maltophilia*. On the other hand, the isolation and study of such effectors hold the potential to develop novel antimicrobial drugs for the targeted therapy of infections caused by *Pseudomonas* and *Escherichia*.

Apart from providing a competitive advantage to *S*. *maltophilia* in polymicrobial communities, probably by increasing its fitness, the VirB/D4 T4SS effectors have another essential function: they inhibit apoptosis in infected lung epithelial cells but induce apoptosis in infected macrophages (Nas et al., 2019).

The Type VI Secretion System (T6SS) is a protein secretion nanomachine utilized by Gram-negative bacteria to deliver toxic effectors into target cells in a contact-dependent manner (Mougous et al., 2006; Perault et al., 2020; Crisan and Goldberg, 2022). Protein effectors exert their toxicity on the bacterial cell envelope and can degrade the peptidoglycan layer and lipid membranes, form pores and interfere with protein synthesis in the cytoplasm of competitor bacteria (Russell et al., 2011, 2013; Ahmad et al., 2019; Nolan et al., 2021). Additionally, T6SS effectors hinder host cell functions, facilitate immune evasion, thereby promoting a successful infection (Hachani et al., 2016), and participate in bacterial metal uptake by assisting low- and high-affinity transport systems in scavenging metal ions from the environment (Wang et al., 2015; Stubbendieck and Straight, 2016; Lin et al., 2017; Si et al., 2017; Han et al., 2019; Yang X. et al., 2021; Li et al., 2022).

Although T6SS genes have been identified in some *S. maltophilia* strains early (Alavi et al., 2014; Bayer-Santos et al., 2019), there is a paucity of experimental evidence demonstrating the function of T6SS effectors in the bacterium. Crisan et al. (2023) reported that the *S. maltophilia* STEN00241 clinical isolate possesses an active T6SS under standard laboratory conditions and the T6SS contributes to the elimination of some heterologous bacterial species. In particular, STEN00241 killed *Burkholderia cenocepacia* strain K56-2 and *E. coli* DH5α in a T6SS-dependent manner, but not *P. aeruginosa* PA14 laboratory strain, the *P. aeruginosa* CF isolate (PA32), and the *S. aureus* JE2. This selectivity in the mode of interspecific interaction within multi-species communities (elimination, competitive co-existence, or hypothetical symbiosis) suggests that the T6SS secretory function is also regulated by various environmental factors. The putative T6SS secretion triggers may be signals generated by the QS of the neighboring bacteria or their various metabolites (Lesic et al., 2009; Lin et al., 2017).

Concluding the chapter on *S. maltophilia* secretion systems, at least one intriguing question remains unanswered: what is the benefit to the bacterium of using both VirB/D4 T4SS and T6SS to produce functionally similar antibacterial proteins? Suggesting that it is not redundancy, the functions of these effectors and/or their trigger mechanisms are thought to be different and need to be further investigated.

To summarize, it should be noted that although five types of secretion systems (type I, II, IV, V, and VI) have been revealed in *S. maltophilia* genomes, further research is needed to fully comprehend their functional roles and potential interactions between the systems.

## Biofilms

The ability of *S. maltophilia* to form biofilms on abiotic surfaces and host tissues is an important VF that plays a crucial role in HAI and multibacterial infections and dramatically decreases the therapeutic efficacy of important antibiotics, including aminoglycosides, fluoroquinolones, and tetracycline (Di Bonaventura et al., 2004, 2007; Pompilio et al., 2010; Sun et al., 2016). Biofilms provide protection to the members of bacterial communities from exposure to antibiotics by reducing their diffusion (Tseng et al., 2013) and increasing their inactivation (Amanatidou et al., 2019). Besides, the biofilm polymer matrix gives bacteria protection from various forms of environmental stress, such as dehydration, UV exposure, salinity, and toxic metals (Hall-Stoodley et al., 2004). High cell density within biofilms and increased oxidative stress result in an elevated mutation rate and enhanced horizontal gene transfer (HGT) (Driffield et al., 2008). Compared to their planktonic counterparts, bacteria in biofilms exhibit greater resistance to nutrient starvation, pH fluctuations, and oxygen radicals (Jefferson, 2004). Biofilms may also increase the level of resistance by altering the expression of pre-existing antibiotic resistance genes (ARGs) (Høiby et al., 2010) as well as the proportion of tolerant or persister cells within the population due to a reduction in bacterial metabolic activity within the biofilm interior (Walters et al., 2003; Wood et al., 2013).

The formation of persister cells is also hypothetically possible due to a reduction in antibiotic concentration within biofilms, since it has been demonstrated that sub-MIC (minimum inhibitory concentration) levels of various antibiotics can induce persister cell formation (Dörr et al., 2009; Johnson and Levin, 2013; Kwan et al., 2013). Nutrient limitation within biofilms perhaps also affects the bacterial stringent response, where (p)ppGpp (alarmone) levels lead to slower bacterial growth and promote the formation of persister cells (McCall et al., 2019; Ro et al., 2021). In addition, biofilms protect bacteria from the host’s immune response by acting as a physical barrier, helping bacteria avoid detection and phagocytosis, and by activating response regulators, genetic switches, or suppressors that affect the activity of immune cells (Hall-Stoodley et al., 2004; González et al., 2018).

The initial stage of the biofilm formation process occurs within the first 30–60 min when planktonic cells adhere to a surface through weak and reversible interactions mediated by semiflexible fimbriae and flagella filaments. The second stage typically begins 4 h later, during which bacterial cells irreversibly attach to and colonize a surface using flagella, pili, and other surface appendages. After adhering to a surface, the cells initiate the production of extracellular polymeric substances, thereby forming a self-secreted polymer matrix of exopolysaccharides, DNA, and proteins (Flores-Treviño et al., 2019). The first microcolonies are generated by the aggregation of cells after approximately 10 h. The third stage occurs in 18–24 h when the biofilm turns into a mature phase. A mature biofilm possesses microchannels to transport water, nutrients, and debris; and bacterial cells within the biofilm express specific genes involved in QS (see below), EPS, and protein production. In mature biofilms, individual or clustered biofilm cells can detach, disperse, and colonize new niches within less than 24 h (de Kievit, 2009; Sun et al., 2016; Flores-Treviño et al., 2019).

At least several putative genes related to *S. maltophilia* biofilm formation have been identified. These biofilm-associated genes include: *spgM* (a biofunctional enzyme with phosphoglucomutase and phosphomannomutase activity); *rmlA* (glucose-1-phosphate thymidylyltransferase); *rmlC* (an epimerase RmlC, also named RfbC); *xanB* (a bifunctional enzyme, phosphomannose isomerase-GDP-mannose pyrophosphorylase); and *rpfF* (cis-11-methyl-2-dodecenoic acid, or synthase for the diffusible signal factor, DSF) (Köplin et al., 1992; Di Bonaventura et al., 2004; Huang et al., 2006; Pompilio et al., 2011; Zhuo et al., 2014). In addition to the genes listed above, numerous genes associated with the synthesis of LPS, fimbria, flagella, and pili, as well as the intracellular c-di-GMP level contribute to biofilm formation (see the corresponding chapters). For instance, almost all (30/31; 97%) *S. maltophilia* isolates harboring *smf*-1, which encodes the fimbrial protein Smf-1, were able to form biofilms (Gallo et al., 2016). The *macABCsm* and *smeYZ* genes, encoding pumps, have also been identified as essential for biofilm formation (Huang et al., 2006; Lin Y.-T. et al., 2014; Lin et al., 2015). An extended list of genes potentially associated with biofilm production can be found in the review by Flores-Treviño et al. (2019).

Recently, Strateva et al. (2023) analyzed 220 *S. maltophilia* strong biofilm producers and found the overall frequency of three biofilm-associated genes as follows: *spgM—*98.6%, *rmlA—*86%, and *rpfF—*66.5%. Meanwhile, Zhuo et al. (2014) have noted that, although the *rmlA*, *spgM,* or *rpfF* are closely related to biofilm formation, they do not significantly affect the average amount of biofilm.

Ramos-Hegazy et al. (2020) analyzed a transposon mutant library for mutations leading to altered biofilm formation. The authors identified the *gpmA* gene, which encodes a glycolytic enzyme, phosphoglycerate mutase, mediating the initial stages of *S. maltophilia* attachment to abiotic surfaces as well as immortalized CF-derived bronchial epithelial (CFBE) cells. The *S. maltophilia* isogenic mutant Δ*gpmA* exhibited a significant decrease in initial attachment and early biofilm formation on polystyrene plates compared to the wild type within the first 2–4 h. Interestingly, after 6 h, there was no difference in biofilm formation between the wild and mutant strains, suggesting that *gpmA* is involved only in the early phase of adhesion and biofilm formation (Ramos-Hegazy et al., 2020; Di Bonaventura et al., 2023).

Pompilio et al. (2020) have analyzed 85 *S. maltophilia* strains isolated from patients with CF and other infections and revealed that over 88% of the isolates were able to form biofilm, with non-CF strains being significantly more efficient compared to CF strains. Meanwhile, the prevalence of the multidrug-resistant phenotype was higher in CF isolates in contrast to non-CF ones (90% vs. 67%). *S. maltophilia* strains susceptible to piperacillin/tazobactam or meropenem produced significantly increased biofilm biomass compared to resistant strains. The authors suggested that susceptible bacteria may utilize biofilms as an alternative defense strategy to evade antibiotic action and to survive within the host (Pompilio et al., 2020).

Liu et al. (2017) have demonstrated the role of a regulatory protein mentioned above, BsmR, an EAL-domain-containing phosphodiesterase, in controlling biofilm formation and swimming motility in *S. maltophilia*. An increase in BsmR expression led to a significant increase in bacterial swimming motility and a decrease in cell aggregation. Thus, BsmR was identified as a negative regulator of biofilm development that degrades c-di-GMP through its EAL domain, thereby activating the expression of a transcriptional regulator, FsnR (see above), which positively controls the transcription of flagellar genes involved in swimming motility (Liu et al., 2017; Zhang et al., 2022).

An outer membrane protein, Ax21, secreted within OMVs and associated with a VF related to QS, is also implicated in biofilm formation (Ferrer-Navarro et al., 2013; An and Tang, 2018). Deletion of *ax21 (Smlt0387)* has been shown to reduce motility, biofilm formation, virulence to larvae of *Galleria mellonella*, tolerance to tobramycin, as well as alter the expression of some genes associated with virulence or antibiotic resistance (Ferrer-Navarro et al., 2013; An and Tang, 2018).

Most interestingly, the analysis of transcriptome profiles of seven clinical *S. maltophilia* isolates, combined with differential gene expression of biofilm vs. planktonic cells, revealed that a relatively small set of shared and commonly regulated genes is involved in the biofilm lifestyle: only about 9.5% of all genes were differentially regulated. On average, approximately 7.5% of all genes were upregulated, and about 2% of all genes were downregulated in biofilms compared to planktonic cells (Alio et al., 2020).

A comprehensive analysis of all available data on the role of various factors in the transition of bacterial cells from a planktonic to a sessile lifestyle in biofilms shows that this transformation is initiated and regulated by many mechanisms that require further study.

## Efflux pumps and virulence

Historically, efflux pumps have been considered to be among the mechanisms that provide bacteria with resistance to antimicrobials. Efflux pumps significantly contribute to the intrinsic antimicrobial resistance of *S. maltophilia*. However, as noted and discussed below, some types of efflux pumps possess an extended range of functions beyond the scope of “antibiotic resistance”, and these pumps are involved in the molecular mechanisms of bacterial virulence.

The genome of *S. maltophilia* contains a formidable arsenal of pumps belonging to various families. This includes ATP-binding cassette (ABC) pumps, MacABCsm (Lin C.-W. et al., 2014) and SmrA (Al-Hamad et al., 2009); Major Facilitator family (MFS) pumps, EmrCAB (Huang et al., 2013) and MfsA (Srijaruskul et al., 2015; Dulyayangkul et al., 2016); a fusaric acid efflux pump, FuaABC (Hu et al., 2012); and eight Resistance Nodulation Division (RND) pumps [SmeABC (Li et al., 2002), SmeDEF (Alonso and Martínez, 2000; Zhang et al., 2001; García-León et al., 2014), SmeGH (Blanco et al., 2019; Li et al., 2019), SmeIJK (Huang et al., 2014), SmeMN (Crossman et al., 2008), SmeOP (Lin C.-W. et al., 2014), SmeVWX (Chen et al., 2011; García-León et al., 2015), and SmeYZ (Lin et al., 2015)].

The efflux pumps encoded in the *S. maltophilia* genome are involved in the removal of a wide spectrum of toxic substances, including antibiotics. The ABC multidrug efflux pump, SmrA, contributes to the elimination of fluoroquinolones, tetracycline, and doxorubicin (Al-Hamad et al., 2009) while MacABCsm, another member of the same family, removes aminoglycosides, macrolides, and polymyxins (Lin C.-W. et al., 2014). The MFS efflux pump, EmrCABsm, facilitates the removal of nalidixic acid, erythromycin, carbonyl cyanide 3-chlorophenylhydrazone, and tetrachlorosalicylanilide (Huang et al., 2013). The fusaric acid tripartite efflux pump, FusA, is involved in the elimination of fusaric acid (Hu et al., 2012). The role of seven out of the eight RND efflux pumps (SmeABC, SmeDEF, SmeGH, SmeIJK, SmeOP, SmeVWX, and SmeYZ) in the antibiotic resistance has been also identified, except for SmeMN (Gil-Gil et al., 2020). Additionally, some RND pumps are involved in the efflux of chloramphenicol, tetracycline, macrolides, quinolones, sulfamethoxazole, trimethoprim, and trimethoprim–sulfamethoxazole (Alonso and Martínez, 2000; Sánchez and Martínez, 2018; Wu et al., 2019). The contribution of pumps to antimicrobial resistance is considered in detail in some comprehensive reviews (e.g., Menetrey et al., 2021; Chauviat et al., 2023).

It is noteworthy that efflux pumps SmeYZ, SmeDEF, and MacABCsm, besides their primary function of removing xenobiotics from bacterial cells, also impact motility, flagella formation, and biofilm development (Lin Y.-T. et al., 2014; Lin et al., 2015; Kim et al., 2018). Lin Y.-T. et al. (2014) demonstrated that a *ΔsmeYZ* mutant was unable to form flagella, resulting in a lack of motility, and exhibited reduced biofilm formation. The SmeYZ pump has been reported to contribute to a number of other physiological functions, including oxidative stress susceptibility, swimming, and, along with the SmeDEF pump, protease secretion (Lin et al., 2015; Wu et al., 2016; Kim et al., 2018). Blanco et al. (2019) reported that SmeGH is also involved in biofilm formation: a Δ*sme*H mutant exhibited an elevated ability to produce a biofilm.

SmeYZ and SmeDEF are thought to be utilized by *S. maltophilia* against eukaryotes. Overexpression of SmeDEF in the *S. maltophilia* strain D457R led to reduced virulence against the social amoeba *Dictyostelium discoideum* (Alonso, 2004), and the loss of SmeYZ decreased *in vivo* virulence in a murine model and increased susceptibility to human serum and neutrophils (Lin et al., 2015). The above evidence significantly supports the suggestion that these RND pumps contribute to the *S. maltophilia* virulence.

The *S. maltophilia* MacABCsm differs from the MacAB homologs of other bacteria (Lin Y.-T. et al., 2014). In particular, the pump possesses its own cognate outer membrane protein (OMP), MacCsm; and the *macABCsm* operon is intrinsically expressed. Additionally, MacABCsm has a wider substrate range for extruding macrolides, aminoglycosides, and polymyxins compared to MacAB-TolC of *E. coli* (Lin Y.-T. et al., 2014).

Another noteworthy function of efflux pumps was described by Wu C.-J. et al. (2022). They revealed that the SmeYZ, SmeDEF, and SbiAB pumps along with other mechanisms, impact the secretion of the siderophore stenobactin and the utilization of iron ions (see below) (Wu C.-J. et al., 2022).

The SmeIJK efflux pump of *S. maltophilia* has been reported to be involved in cell envelope integrity and the envelope stress response. Huang et al. (2014) demonstrated that a smeIJK-deleted mutant has increased sensitivity to membrane-damaging agents (MDAs) compared to the wild-type strain and exhibited an increased RpoE-mediated envelope stress response. In addition, sublethal MDAs concentrations induced *smeIJK* expression in an RpoE-dependent manner.

Summarizing the above, the analysis of current data on efflux pumps suggests that the historically held belief regarding their main functions should be reevaluated, and bacterial efflux pumps are much more than antibiotic resistance determinants. Since pumps are revealed in both clinical and environmental strains (Youenou et al., 2015) and considering the environmental origin of *S. maltophilia*, the functions of efflux pumps may be linked to bacterial physiology and adaptation to various niches and environments, as well as coexistence within complex multi-species communities.

## Virulence and iron

Iron is vital for the growth and proliferation of non-fermenting Gram-negative bacilli, including *S. maltophilia*. Competition for iron ions between bacteria and the host during chronic infections can be detrimental to the host. Bacterial iron uptake may lead to local tissue damage and systemic dysfunction, e.g., anemia of inflammation, also known as anemia of chronic disease, observed in infectious, inflammatory, autoimmune, neoplastic, and chronic kidney diseases (Jurado, 1997). Iron plays a role in bacterial pathogenicity and host defense mechanisms, which is often underestimated. In *S. maltophilia*, iron limitation induces biofilm formation, increases EPS production, and reduces the generation of reactive oxygen species (ROS) (Kalidasan et al., 2018). Therefore, bacterial systems aimed at acquiring and transferring iron ions into bacterial cells are considered significant VFs.

**Figure 2:**
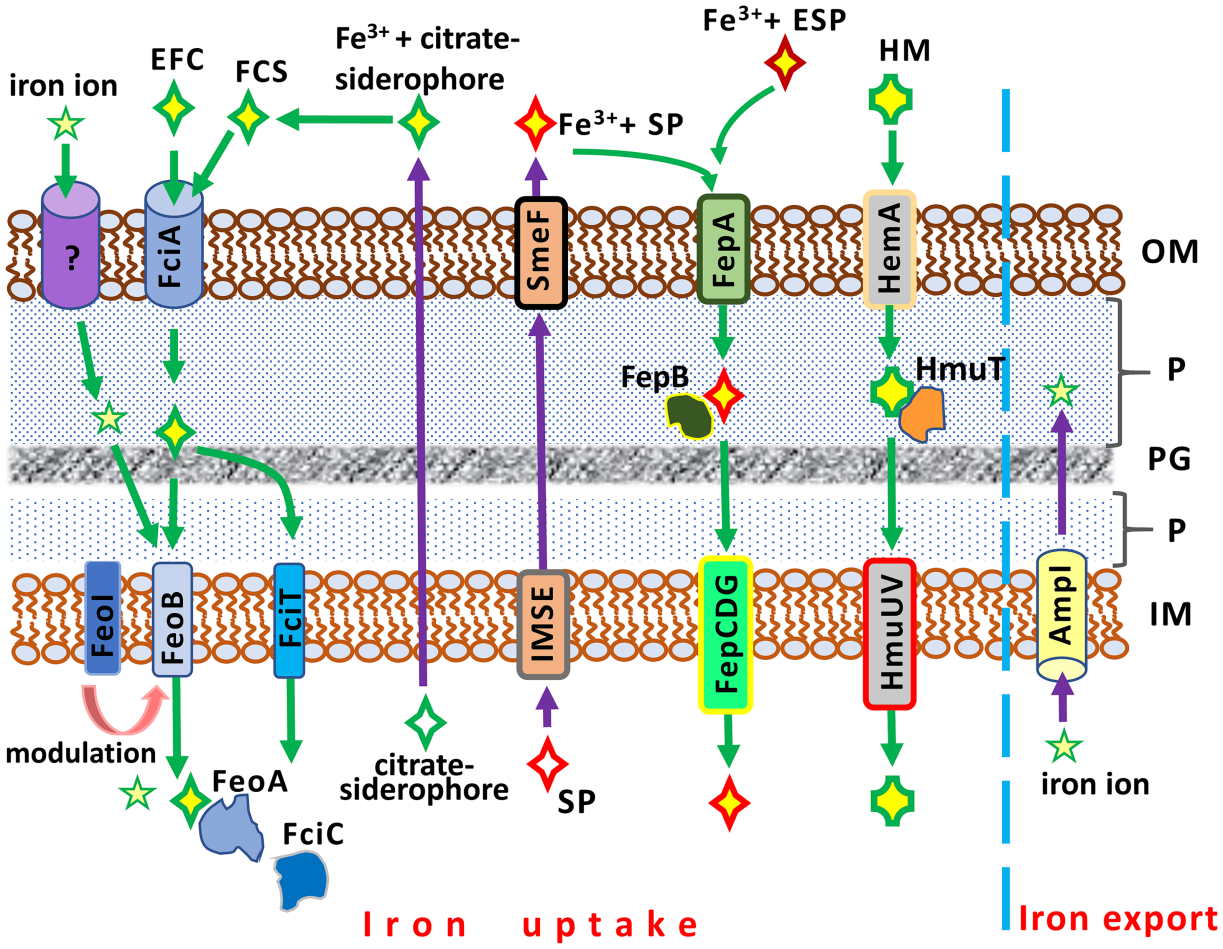
Iron acquisition and iron export systems in *Stenotrophomonas maltophilia*. IM, inner membrane; P, periplasm; PG; peptidoglycan; OM, outer membrane. AmpI, the inner membrane iron exporter; EFC, exogenous ferric citrate; ESP, an exogenous siderophore (siderophores produced by other bacteria, e.g., *Pseudomonas aeruginosa*); FciA, the outer membrane receptor for ferric citrate uptake; FciT (inner membrane protein) and FciC (cytoplasmic protein), putative proteins for citrate-mediated iron acquisition; FCS, the ferric citrate siderophore complex; FeoA, a cytoplasmic protein; FeoABI, the inner membrane transporter system of ferric citrate; FepA, the ferri-siderophore uptake system; FepB, a protein delivering ferric siderophore from the periplasm; FepCDG, the inner membrane transporter system for ferric siderophores; HemA, the TonB-dependent outer membrane receptor for hemin; HM, hemin; HmuT, a hemin-transporting protein; HmuUV, the inner membrane hemin transporter; IMSE, the inner membrane siderophore exporters (EntS for enterobactin, SmeY, SbiA, and SmeD for stenobactin); SmeF, the outer membrane exporter for siderophore; SP, *S. maltophilia* siderophore (stenobactin or enterobactin).

While a basic understanding of iron uptake in Gram-negative bacteria has been achieved, many molecular mechanisms involved in this process in *S. maltophilia* remain unclear. Similar to other bacteria, *S. maltophilia* possesses a number of iron acquisition mechanisms that exhibit functional redundancy (Figure 2).

Two distinct iron uptake pathways are encoded by the *S. maltophilia* genome. These pathways include a siderophore- and a heme-mediated acquisition system (Kalidasan et al., 2018). The *entAFDBEC* operon controls the synthesis of the siderophore enterobactin, which belongs to the class of catecholamines, binding and transporting Fe^3+^ into the bacterial cell. The heme-mediated uptake system is under the control of the *hgbBC* and probably also the *hmuRSTUV* operons (Adamek et al., 2014; Kalidasan et al., 2018).

The simplest and least efficient absorption system is based on the diffusion of iron ions across OMPs (Liao et al., 2022). A more advanced iron uptake system employs siderophores, high-affinity iron-chelating molecules, to capture iron ions from the environment. These iron-siderophore complexes are recognized by specific OMPs and transported to the cytoplasm. *S. maltophilia* strains can produce at least two catecholate siderophores, stenobactin and an EntC-dependent catecholate siderophore which is sufficiently similar to, but distinct from, enterobactin of enteric bacteria (Nas and Cianciotto, 2017; Zhang et al., 2021; Wu C.-M. et al., 2022). Although one previous study (Jurkevitch et al., 1992) reported that *S. maltophilia* produces pseudobactin for iron uptake, this finding has not yet been confirmed. The export of siderophores is mediated by a system of membrane transport proteins (EntS, SmeY, SbiA, SmeD) of the inner membrane and TolC-exporter (SmeF for stenobactin) of the outer membrane (Wu C.-M. et al., 2022).

The ferric-enterobactin complex is recognized and taken up by the TonB-dependent FepA (ferric enterobactin protein), the enterobactin receptor on the outer membrane. Subsequently, FepB, the periplasmic binding protein, transports the iron-siderophore complex from the periplasm to the cytoplasm via the FepCDG transporter (Faraldo-Gómez and Sansom, 2003; Kalidasan et al., 2018).

Interestingly, while competing for iron, *S. maltophilia* can demonstrate cheating by using exogenous siderophores of xenogeneic origin. It has been reported that *S. maltophilia* can facilitate the uptake of ferri-pyochelin from *P. aeruginosa* in an iron-depleted condition (Pan et al., 2022).

An alternative to the catecholate siderophore is the citrate siderophore, which can be produced by *S. maltophilia* or obtained from exogenous sources. The Fe^3+^-citrate-siderophore complex is transported into the cytoplasm through a system of specific outer and inner membrane proteins (Figure 2). FciA (Smlt1148) has been reported as the main receptor for ferric citrate acquisition in *S. maltophilia* KJ (Liao et al., 2022).

Another pathway of iron acquisition in *S. maltophilia* is the heme-mediated uptake system, which utilizes its specific transporters. The putative outer membrane receptor for hemin is HemA, which is under negative control of the predicted transcriptional factor HemP (Shih et al., 2022). Hemin uptake is controlled by the *hemP-hemA-smlt0796-smlt0797*, *hgbBC,* and potentially *hmuRSTUV* operons (Kalidasan et al., 2018; Shih et al., 2022). The *hemP-hemA-smlt0796-smlt0797* operon, in turn, is negatively regulated by the ferric uptake regulator Fur (see below) (Shih et al., 2022).

Iron uptake in *S. maltophilia* is balanced by the opposite function of iron export. Excess free iron ions in the cytoplasm, which can be toxic to bacterial cells and potentiate ROS toxicity, are removed from the cell by the inner membrane iron exporter, AmpI (Huang et al., 2019). In an iron-depleted condition, AmpI function is inhibited, resulting in iron storage in the *S. maltophilia* cytoplasm. Notably, AmpI also allows *S. maltophilia* to reduce β-lactam-induced stress: the expression of *ampI* increases significantly upon exposure to β-lactams (Huang et al., 2019).

The iron uptake system in Gram-negative bacteria is under the control of many regulators. The key iron-dependent regulator of iron acquisition is the ferric uptake regulator protein (Fur) (Escolar et al., 1999), which is also involved in virulence and protection against oxidative stress (Carpenter et al., 2009). In the presence of Fe^2+^, Fur forms a complex with Fe^2+^ ions that binds to the Fur boxes of bacterial DNA, thereby inhibiting the transcription of iron transport genes (Bagg and Neilands, 1987; Escolar et al., 1999; Lee and Helmann, 2007). In the absence of iron, Fur releases its repressor effect, and genes involved in iron transport are expressed.

Interestingly, impairing Fur in *S. maltophilia* may increase its virulence. The spontaneous *fur* mutant, derived from the wild-type strain *S. maltophilia* K279a exhibited increased virulence in the *G. mellonella* killing assay compared to the parental wild-type K279a strain (García et al., 2015).

Other iron-regulating systems and putative regulators have also been identified in *S. maltophilia*. The FciTABC and FeoABI systems are responsible for ferric citrate utilization under iron-depleted conditions. Ferric citrate is taken up by FciA, a TonB-dependent OMP, and then transported across the inner membrane through the FeoABI pathway. The genes *fciA, fciT,* and *fciC* contribute to ferric citrate acquisition and are under the control of the *fciTABC* operon (Liao et al., 2022). AmpR, a transcriptional regulator in *S. maltophilia,* regulates stenobactin synthesis in an iron-depleted condition, however, its contribution to the acquisition and utilization of ferri-stenobactin and ferric citrate is thought to be insignificant (Liao et al., 2020). The uptake of hemin (as mentioned above) is under the control of the *hemP-hemA-smlt0796-smlt0797*, *hgbBC,* and potentially *hmuRSTUV* operons (Kalidasan et al., 2018; Shih et al., 2022). RNA polymerase sigma factors may also be involved in iron regulation in *S. maltophilia* (Kalidasan et al., 2018).

The iron uptake system is considered not only a virulence factor, but also a regulator of other VFs. Specifically, *S. maltophilia* K279a produced denser biofilms, and higher levels of EPS and DSF in an iron-depleted condition. In addition, the strain exhibited greater virulence than that cultivated under normal nutritional or iron-rich conditions (García et al., 2015; Kalidasan et al., 2018). Clinical and environmental *S. maltophilia* isolates grown under iron-limited conditions demonstrated increased nematocidal activity against *Caenorhabditis elegans* and increased siderophore production (Azman et al., 2019). There have also been contradictory observations: in particular, Jurado (1997) has reported that excess iron can enhance virulence in bacteria. Surplus iron ions have also been shown to upregulate RTX family toxin genes, including the putative virulence gene *frpA*, and downregulate *frpC* in *S. maltophilia* (Adamek et al., 2014).

Probably, fully understanding the influence of high/low levels of iron on *S. maltophilia* virulence requires further research, and the complexity of the iron regulatory system as well as its potential interaction with other bacterial metabolic global regulators should be taken into account. One potential avenue for further research could be the identification of the iron impact on virulence in the context of the development of a novel class of antibiotics, conjugates of a synthetic cephalosporin with an artificial siderophore. In response to the introduction of the new antibiotic, it has been reported the emergence of resistance to cefiderocol is associated with mutations in the iron uptake system genes (Werth et al., 2022). In this regard, the question arises: how will virulence change in cefiderocol-resistant isolates? An experimentally confirmed answer to this question may have clinical relevance.

## Quorum sensing system

Similar to most Gram-negative bacteria, *S. maltophilia* possesses quorum sensing (QS), a signaling mechanism through which bacterial cells communicate to exchange information about cell density and adjust their gene expression accordingly (Huang and Lee Wong, 2007b). The system is responsible for the production of extracellular signaling molecules known as autoinducers, their detection, and initiating the bacterial response to the appearance of these molecules in the environment. Autoinducers accumulate in the environment, and when their concentration reaches a certain threshold, nearby bacteria are able to detect them. Through the exchange of signaling molecules, cells regulate their metabolic mechanisms related to colonization and virulence, including alterations in motility, biofilm formation, production of extracellular effectors, competition, and resistance properties (Huedo et al., 2014; Papenfort and Bassler, 2016; Paul et al., 2018; Yero et al., 2020).

Genome analyses have revealed that *S. maltophilia* does not synthesize *N*-acyl homoserine lactones (*N*-AHLs) or autoinducer 2 (AI2), signaling molecules typically found in other Gram-negative bacteria (Zhu et al., 2001; Vfselova et al., 2003). The main QS signaling molecule produced by *S. maltophilia* is known as DSF, represented by *cis*-Δ2-11-methyl-dodecenoic acid, an unsaturated fatty acid, and seven of its structural derivatives. The synthesis of DSF molecules is controlled by the *rpfF* and *rpfB* genes (Huang and Lee Wong, 2007b). The *rpf* (regulation of pathogenicity factors) gene cluster encodes RpfF synthase, fatty Acyl-CoA ligase, and the two-component RpfC/RpfG system responsible for the perception and transduction of DSF (Huang and Lee Wong, 2007a; Bi et al., 2014). Activated RpfF synthase converts c-di-GMP into a linear nucleotide pGpG or two GMP molecules, thereby regulating the expression of genes related to motility, biofilm formation, and virulence in *rpfF*-1 strains (Huedo et al., 2019).

Two variants of the *rpf* gene cluster, *rpf*-1 and *rpf*-2, have been found in *S. maltophilia* (Huedo et al., 2014). Notably, strains belonging to different *rpf* types exhibit distinct genotypic and phenotypic characteristics. *Rpf*-1-type *S. maltophilia* strains can produce DSF in response to various environmental signals, while *rpf*-2-type strains, with a truncated sensory region in the N-terminus of RpfF synthase, activate their DSF production only after detecting exogenous DSF (e.g., from other bacteria or *S. maltophilia* strains of the *rpf*-1 type). Therefore, only *rpf*-1-type strains can control biofilm formation, as well as the motility and virulence in surrounding bacteria (Huedo et al., 2014, 2015).

It is noteworthy that *rpf*-1-type *S. maltophilia* strains, particularly those in genogroup C, exhibit higher resistance to colistin and increased virulence against *G. mellonella*. Meanwhile, this association was not revealed in another model using the nematode *C. elegans* (Yero et al., 2020).

Thus, genotyping and identifying the *rpf* type appear to be useful and important tools for epidemiologic surveillance, considering the potential exchange of *rpf* clusters among *S. maltophilia* strains through recombination during horizontal gene transfer (Huedo et al., 2015).

*Stenotrophomonas maltophilia* has been found to possess a two-component signal transduction system (TCS) called BfmA–BfmK (Smlt4209–Smlt4208). The BfmA transcription factor, a component of the TCS, binds to the *bfmA–bfmK* promoter region and *Smlt0800* (*acoT*), a gene encoding acyl-coenzyme A thioesterase, associated with biofilm formation (Zheng et al., 2016).

Coves et al. (2023) reported a putative TetR-like transcriptional regulator (locus tag SMLT2053) involved in biofilm formation in the K279a strain. The regulator controls its own β-oxidation operon (*Smlt2053*-*Smlt2051*) and is capable of sensing free long-chain fatty acids from the environment, including the QS signal DSF.

The transcriptional regulator AmpR, previously mentioned as a regulator of stenobactin synthesis under iron-deficient conditions, also affects the production of DSF and QS-dependent VFs. Alcaraz et al. (2022) have reported the negative impact of AmpR on biofilm production, as well as on architecture and matrix polysaccharides production in *S. maltophilia*. The *ampR* deficient mutant K279a *ampR^FS^* exhibited the highest adherence in tubes, the highest biomass production in microplates, and formed biofilms with improved thickness. In contrast, the strain with a constitutively active AmpR, K279aM11, was the least efficient in biofilm formation (Alcaraz et al., 2022). Given that AmpR is a positive regulator of β-lactam-induced β-lactamase expression (Okazaki and Avison, 2008) and iron depletion-mediated stenobactin synthesis (Liao et al., 2020), it is hypothetically possible that *S. maltophilia* may compensate for the loss of AmpR activity by enhancing biofilm and polysaccharides production to survive under adverse conditions.

It is worth noting that, in contrast to *P. aeruginosa*, *S. maltophilia* lacks a complete canonical LuxI/LuxR QS system utilizing acyl-homoserine lactones (AHL) as signaling molecules (see above). However, Martínez et al. (2015) revealed through comparative genomic analysis that *S. maltophilia* has a LuxR-like gene, *Smlt1839*, encoding the SmoR regulator (*Stenotrophomonas maltophilia* orphan regulator), which *in vitro* binds the synthetic lactone 3OC_8_-HSL, a natural analog of which is produced by *P. aeruginosa*. Adding concentrated supernatant from the medium on which lactone-producing *P. aeruginosa* was cultured provided an impetus to increase the swarming motility of *S. maltophilia* on Petri dishes. In other words, although *S. maltophilia* lacks the canonical LuxI/LuxR system, it is able to recognize QS signaling molecules of other species with the LuxI/LuxR system through its homologous intercellular exchange system. It is hypothetically possible that this system is linked to the T6SS and can, under certain conditions, induce the secretion of effectors to inhibit competitor growth (see above).

At the same time, *S. maltophilia* and *P. aeruginosa* can coexist and grow together in polymicrobial biofilms, e.g., in the lungs of CF patients (Ryan et al., 2008). Such a symbiotic (temporarily symbiotic or deferred competitive?) coexistence affects their susceptibility to antibiotics. Within two-species biofilms, *S. maltophilia* produced the DFS that was detected by the two-component sensor BptS in *P. aeruginosa*. As a result, the latter decreases its susceptibility to polymyxin B and colistin compared to *P. aeruginosa* monospecies biofilms (Ryan et al., 2008).

In considering the DSF system in *S. maltophilia*, it is worth noting the phenomenon of secretion through OMVs (Ferrer-Navarro et al., 2013). OMVs are small nanostructures secreted by bacteria, that can transport nucleic acids, proteins, and various small molecules, such as β-lactamases, to the surrounding environment. Devos et al. (2015) have found that *S. maltophilia* dramatically increased its vesicle secretion in the presence of imipenem. Of particular interest was the composition of the molecules transported by the OMVs: two types of β-lactamases encoded by chromosomes, OMPs, and flagellins Smlt0387 and Smlt0184 (Devos et al., 2015). These flagellins are homologous to Ax21, a protein involved in motility and biofilm formation in *Xanthomonas oryzae*. The functional role of this protein in *S. maltophilia* has not been determined, but it is thought that its secretion is initiated by DSF. Recently, the secretion system via OMVs is considered a potential VF, based on data demonstrating its effect on motility and biofilm formation in *X. oryzae* (Park et al., 2014).

## Virulence and bacteriophages

The association between virulence and the presence of filamentous phage genes in the *S. maltophilia* genome was initially reported in 2006 (Hagemann et al., 2006). Among 47 *S. maltophilia* strains of clinical and environmental origin, Hagemann et al. (2006) identified five isolates bearing the *pI* gene of the filamentous phage phiLf, which was identical to the *zot*-like gene encoding the zonula occludens toxin in *V. cholerae*. In addition to the *zot*-like gene, six other genes related to a phage life cycle were identified in the *S. maltophilia* genomes. The authors suggested that phage genes could be transferred between strains via mobile genetic elements, potentially increasing the virulence of *S. maltophilia*.

Another mechanism through which filamentous phages vicariously increase the virulence of Gram-negative bacteria has been reported. It has been observed that the extracellular matrix produced by *P. aeruginosa* self-assembles into a liquid crystalline structure together with filamentous Pf bacteriophages in CF sputum (Secor et al., 2016). These liquid crystals enhance biofilm function by increasing adhesion and preventing the diffusion of antibiotics through biofilms, thereby contributing to increased antibiotic tolerance (Secor et al., 2015, 2016).

No previous studies have reported a similar virulence mechanism in CF respiratory *S. maltophilia* isolates, and confirmation of this VF, as well as a putative novel form of symbiosis between bacteria and phages, holds potential for further research.

The role of phages in increasing bacterial virulence has been extensively studied (Wagner and Waldor, 2002; Brüssow et al., 2004), however, bacteriophages may also provide a selective pressure against bacteria expressing specific VFs (León and Bastías, 2015). If the phage receptor coincides with a VF, such as lipopolysaccharide or type IV pili, its modification through mutations results in decreased virulence and reduced fitness (León and Bastías, 2015). McCutcheon et al. (2018) have reported that the type IV pilus is the primary receptor for DLP1 and DLP2 bacteriophages that are able to infect both *S. maltophilia* and *P. aeruginosa*. Deletion of the primary pilin subunit by inactivation of *pilA* in *S. maltophilia* prevented phage binding and subsequent lysis by both bacteriophages, while the mutant strain exhibited reduced virulence. This phenomenon can be thought of as a fitness cost: when a bacterium acquires properties that are beneficial for its specific living conditions, it must compensate for the loss of other, less important properties.

## Conclusion

In recent decades, there has been considerable interest in understanding the mechanisms underlying the virulence of *S. maltophilia*. The intrinsic multidrug resistance of the bacterium, its ability to rapidly adapt to unfavorable environmental conditions and new habitat niches, and its sophisticated switching of metabolic pathways are features that are attracting the attention of experts studying the fundamental mechanisms of virulence as well as clinical researchers.

When considering the virulent properties of *S. maltophilia*, it is important to keep in mind that the bacterium is characterized by high intraspecific variability: strains isolated in the same hospital and even from the same patient may belong to relatively distant phylogenetic groups and have different phenotypes (Valdezate et al., 2004; Pompilio et al., 2016; Chung et al., 2017). The fast accumulation of adaptive mutations occurring under the selective pressure of hospital conditions or the host cells, as well as horizontal gene transfer, are thought to be possible reasons for such heterogeneity. Understanding the molecular processes that ensure rapid adaptation and, consequently, the survival of the microorganism under adverse conditions will allow the identification of potential targets for the development of novel antibacterial drugs, as well as a better understanding of interspecific interactions in polymicrobial infections and the mechanisms of metabolic switching during the transition of opportunistic pathogens from “natural” lifestyle to infectious intervention.

## Author contributions

VM: Conceptualization, Visualization, Writing – original draft, Writing – review & editing. RH: Formal analysis, Writing – original draft, Writing – review & editing. DZ: Visualization, Writing – original draft, Writing – review & editing. IC: Conceptualization, Writing – original draft, Writing – review & editing.

## Footnotes

^1^ https://lpsn.dsmz.de/
